# The burden of diabetic complications in subjects with type 2 diabetes attending the diabetes clinic of the Obafemi Awolowo University Teaching Hospital, Ile-Ife, Nigeria - a cross-sectional study

**DOI:** 10.11604/pamj.2022.43.148.36586

**Published:** 2022-11-22

**Authors:** Rosemary Temidayo Ikem, Adenike Christianah Enikuomehin, David Olubukunmi Soyoye, Babatope Ayodeji Kolawole

**Affiliations:** 1 Department of Medicine, Obafemi Awolowo University, Ile Ife, Nigeria,; 2 Department of Medicine, University of Medical Sciences Ondo, Nigeria

**Keywords:** Diabetes mellitus, type 2 diabetes, complications of diabetes

## Abstract

**Introduction:**

the increasing prevalence of diabetes (DM) worldwide has resulted in an increase in the morbidity and mortality of DM. This burden is as a result of the development of the chronic complications associated with it. This study determined the burden of occurrence of microvascular and macrovascular complications of subjects with type 2 diabetes attending the out -patient clinic of a tertiary hospital in south west Nigeria.

**Methods:**

this cross-sectional study involved 400 consecutive subjects with type 2 diabetes. A study proforma was used to document the socio demographic data. While clinical assessment for anthropometric measurement, blood pressure was done. Laboratory measurement of blood glucose control and lipids were done. Assessment of the occurrence of microvascular and macrovascular complications were performed and documented.

**Results:**

four hundred type 2 DM participants made up of 190 males and 210 females with a mean age of 60.35±9.53 years, with a mean age of 60.35(SD 9.53) years for males and 60.81(SD10.29) years for females. Median duration of DM for all subjects was 6.00(IQR 3.00 - 11.00) years. Majority (45%) of the participants were overweight. The prevalence of hypertension was 78% and poor glycaemia using HBA1C was 75.5% and 59.8% had dyslipidaemia. The occurrence of microvascular complications (diabetic neuropathy - 82%, diabetic retinopathy - 46% and diabetic nephropathy - 44%) 69.3% while macrovascular complications (peripheral arterial disease - 42.5%, stroke - 4%, electrocardiographic changes if ischaemic heart disease -9.3% and left ventricular hypertrophy - 22%) in 49%. Regression analyses showed advancing age aOR (1.18 [95%CI 1.01 - 1.38]) and waist circumference (aOR 1.17 [95% CI 1.00 - 1.36]), as significant contributors to the presence of diabetes complications.

**Conclusion:**

the risk factors of both microvascular and macrovascular complications remain high in our clinic and this is linked to the high burden of diabetes mellitus and its long duration.

## Introduction

The burden of Diabetes mellitus (DM) is increasing and the worldwide prevalence of DM has risen dramatically over the past two decades. The global prevalence of DM is 9.3% among adults aged 20-79 years, and the number of people living with DM is projected to increase from the present 463 million to 700 million by 2025 [1]. Diabetes Mellitus is an important health problem worldwide, being a major chronic non-communicable condition that causes a significant degree of morbidity and mortality. In Africans, there has been a progressive increase in the prevalence of DM and the burden is expected to increase even further [2]. It has been recognized as a continuing health challenge for the twenty-first century, both in developed and developing countries, due in part to westernization of lifestyles as well as increasing urbanization and economic development [3]. In Nigeria, over the past 30 years the prevalence of Diabetes has been increasing steadily from 0.4% to 59.2% [4-6] and if uncontrolled has been associated with development of complications. The rapidly increasing global burden of DM, coupled with the associated morbidity and mortality, underscores the imperative for continued generation and application of evidence-based approach to reduce risks in these high-risk patients [7]. Chronic hyperglycaemia is associated with an increased risk of micro-vascular complications, as shown in the Diabetes Control and Complications Trial (DCCT) in individuals with type 1 diabetes and in the United Kingdom Prospective Diabetes Study (UKPDS), it is associated with microvascular and macrovascular complications in people with type 2 diabetes [8,9]. It is important to know the prevalence of DM complications, to aid policy formulation, health promotion, and reevaluation of preventive and management strategies. There has been no study in Nigeria that examined the macro- and microvascular complications of DM in a single study. The objective of this study is to determine the pattern and frequency of Type 2 diabetic (T2DM) complications in an out- patient clinic.

## Methods

**Study design and setting:** the study was a cross-sectional descriptive study, conducted at the Diabetes clinic of Obafemi Awolowo University Teaching Hospital Complex (OAUTHC), a tertiary health institution in the south west of Nigeria.

**Study population:** the study population was made up of consecutive Type 2 DM patients aged thirty (30) years and above being followed up at the diabetes medical outpatient clinic of OAUTHC and diagnosed using WHO diagnostic criteria [10]. The sample size was calculated using the formula (Fisher´s formula), N = 384.16, a non-response rate of 5% was assumed, with approximation to 400. Patients who have Type 1 diabetes mellitus, pregnant, on steroids, or unwilling to participate were excluded from the study.

**Data collection:** using a study proforma, the demographic data, duration of DM and duration of hypertension (if present), medications both for DM, hypertension and lipids were obtained. History of physical activities, smoking, alcohol ingestion, numbness of the limb, blurring of vision, nocturia, frothiness of urine, stroke, transient ischemic attack, palpitation and chest pain and compliance with medication were obtained and documented. History of intermittent claudication was taken as evidence for peripheral arterial disease.

### Clinical assessment

(i) The weight and height of the study subjects were measured using standard protocol. The weight was measured in kilogram (kg) to the nearest 0.05kg [11]. The height was measured in meters to the nearest 0.01m. The body mass index (BMI) was calculated using the formular:

BMI = weight (kg)/height (m^2^).

And BMI was classified using World Health Organization guidelines [11].

(ii) Blood pressure was measured using mercury sphygmomanometer with appropriate cuff (encircling at least 75- 80% of the arm) after the patient had rested for at least five minutes in a sitting position. The phase one and five of Korotkoff sounds were used to determine the systolic and diastolic blood pressures respectively. Two measurements were taken and their average taken as the individual´s blood pressure. Study participants were considered to have systemic arterial hypertension if systolic BP ≥ 130 mm Hg or diastolic BP ≥ 85 mm Hg [12].

(iii) Waist circumference was taken in a standing position at the end of a normal expiration, with arms at the side, using a non-elastic measuring tape placed in the mid-point between the iliac crest and the lowest rib in the mid -axillary line. The hip circumference was measured at the maximum circumference around the hips. Waist-to-hip ratio (WHR) > 0.9 in male and > 0.85 in female was defined as central obesity [13,14].

### Definitions-complications of diabetes

**Diabetic neuropathy:** diabetic neuropathy was determined by the symptoms of numbness of the limbs, paresthesia and the absence of vibration sense using a 128-Hz tuning fork) or loss of touch in equal/ greater than three places using a 10g Semmes -Weinstein monofilament. Grades of Neuropathy defined as Grade 0 denotes no symptoms but a sign of neuropathy, grade 1 is > 2 abnormal neurological tests, grade 2 for > 2 abnormal tests plus symptoms, while grade 3 is >2 abnormal tests plus debilitating symptoms [15].

**Diabetic retinopathy (DR):** an ophthalmoscope was used to examine the eyes for the presence of diabetic retinopathy graded as, no apparent DR, mild non-proliferative DR, moderate non-proliferative DR, severe non-proliferative DR, and proliferative DR based on finding the diagnostic signs of retinopathy such as dot and blot haemorrhages, hard exudates, cotton wool spot, neovascularization or maculopathy on fundoscopy (using the new International Clinical Diabetic Retinopathy Diseases Severity Scale) [16]. Diagnosis of retinopathy was based on finding the diagnostic signs of retinopathy on examination of the eye with ophthalmoscope, done by the researchers, and supported by the ophthalmologists.

**Diabetic nephropathy:** patients were considered to have nephropathy if they have microalbuminuria in spot early morning midstream urine collected in a sterile bottle.

**Peripheral arterial disease:** peripheral arterial disease was assessed by palpation of the dorsalis pedis, and posterior tibial pulses and by determining the ankle-brachial index (ABI) using a hand-held Doppler. The ABI was calculated by dividing the higher of the systolic pressure from dorsalis pedis or posterior tibial artery with the higher brachial systolic pressure. PAD was defined as an ABI < 0.9 in at least one leg [17,18].

**Ischaemic heart disease:** was determined by documented angina symptoms such as central chest pain, palpitation, and by performing 12 lead resting ECG. The diagnosis of ischaemic heart disease was made based on the American Heart Association criteria [19]. These criteria include ECG features of significant ST- segment depression defined as an ST-segment depression of more than 1mm in more than one lead, and T-wave inversion. Myocardial infarction was defined as an ST-segment elevation (convex upwards) of more than 0.08 sec, associated with T-wave inversion in multiple leads, and reciprocal ST-segment depression in opposite leads [19]. ECG readings were confirmed by cardiologists.

**Cerebrovascular disease:** cerebrovascular disease was defined by the history of transient ischemic attack or presence of stroke [20].

**Laboratory analysis:** venous blood was drawn under sterile conditions for the following investigations

i) Glycosylated haemoglobin (HbA1c) - using boronate affinity chromatography method. Glycaemic control was based on measurement of HbA1c, HbA1c ≥ 6.5% was considered as diabetic. Good glycaemic control was taken as HbA1c < 7%, and poor glycaemic control was regarded as HbA1c ≥ 7%) [20,21].

ii) Fasting lipid profile - Fasting serum total cholesterol (TC) was measured using the CardioChek® PA lipid Analyzer. Fasting serum triglyceride (TG) concentrations were measured using same method as above. Low-density lipoprotein cholesterol (LDL-c) was calculated by the Friedward equation LDL= (TC- HDL-c) - TG/5. High-density lipoprotein cholesterol (HDL-c) was measured using the CHOL CardioChek® PA lipid Analyzer. TC/HDL-c ratio = the atherogenic factor was calculated by dividing TC by HDL-c. Dyslipidaemia was defined as; LDL) > 100mg/dl (2.6mmol/l); HDL < 40mg/dl (1.2mmol/l) for men; < 50mg/dl (1.3mmol/l) for women; Triglycerides > 150mg/dl (1.7mmol/l) [22].

### Statistical analysis

Data was analyzed on computer using SPSS software (version 23, IBM). Data is represented using descriptive statistics such as tables, for socio-demographics variables. Mean levels, frequencies and percentages were determined. Continuous variables with normal distribution were represented as mean (standard deviation), while continuous variables with skewed distribution were represented with median (interquartile range). The student´s-test was used for comparison of means of continuous variables, while Chi-square was used for categorical variables in participants. P < 0.05 was taken as statistically significant. Univariate and multivariable regression analyses were done to explore factors associated with the presence of diabetic complications. Factors with p-value of less than 0.2 from the univariate analysis, or that were known to be associated with complications of diabetes from previous studies, were selected for analysis into the binary logistic regression.

**Ethical consideration:** the research proposal was approved by the Research and Ethics Committee of the Obafemi Awolowo University Teaching Hospital, Ile - Ife. Informed consent was obtained from all the participants before recruitment.

## Results

### Demographic and social characteristics of the study population

Four hundred (400) type 2 diabetic patients who satisfied the inclusion criteria, were consecutively recruited, for the study. There were 190 (47.5%) males and 210 (52.5%) females. The mean age of the study population was 60.6 (SD 9.93) years with a mean age of 60.35(SD 9.53) years for males and 60.81 (SD10.29) years for females. Their age ranged between 34- 85 years for male and 37- 83 years for female. The median duration of DM for all subjects was 6.00 (IQR 3.00 - 11.00) years. The occupation of majority of the study participants were trading 176 (44.0%), followed by civil servants 70 (17.5%). Most 335 (84.2%) of the study participants never smoked. Of the 63 who smoked, only 2 are current smokers. All subjects with the history of smoking cigarette smoked less than five pack years, only five (2.6%) males smoked more than five pack years. Consumption of alcohol differed between males and females participants with more males (37. 4%) consuming alcohol significantly (p < 0.001). While 8 (4.2%) of males and none of the female consumed more than 60g of alcohol per day.

### Anthropometric profile of the study population (Table 1)

**Table 1:** the anthropometric profile of the study population

Variables	Males (n=190) (Mean +SD)	Females (n=210) (Mean +SD)	p - value
**Weight(kg)**	73.38 (12.39)	72.99 (13.72)	0.762
**Height (m)**	1.67 (0.08)	1.59 (0.06)	<0.001
**WC (cm)**	92.79 (9.90)	94.19 (11.31)	0.001
**HC (cm)**	97.51 (9.65)	104.11 (11.73)	<0.192
**WHR**	0.95 (0.08)	0.91(0.10)	<0.001
**BMI (kg/m2)**	26.31 (4.31)	28.81 (4.99)	<0.001

BMI- Body mass index, WC- Waist circumference, HC- Hip circumference, WHR -Waist to hip ratio

Mean values of body mass index and waist circumference were significantly higher in females than males (p < 0.001) while mean height and waist to hip ratio were statistically significant and higher in males than females (p < 0.001). Overall, 180(45%) study subjects were overweight, making it the predominant category. One hundred and sixteen (29.0%) had normal body mass index, and 101(25.3%) were obese and. Seventy-two (37.9 %) males and 44 (21.1 %) females were of normal BMI. Eighty-seven (45.8%) and 93 (44.3%) were overweight while 28(14.7%) males and 73(34.8%) females were obese. Obesity rate was significantly higher in females than males (p < 0.001).

**Clinical characteristics of study population:** One hundred and seventy-one (42.8%) study participants have had DM for 5 years or less while 109 (27.2%) had DM for more than 10 years. Of the 400 subjects, 312(78%) had history of hypertension. Majority of the study participants 238 (59.5%) had duration of hypertension of 5 or less years, 79 (19.75%) for 6-10years, while 83(20.75%) had hypertension of longer than 10 years.

**Quality of glycaemic control:** ninety-eight (24.5%) of the subjects had good glycaemic control, with HBA1c < 7%, and 302(75.5%) had HBA1c > 7%, p-value 0.001 two hundred and thirty-nine (59.8%) subjects had dyslipidaemia, while 161(40.3%) had normal fasting lipid profile according to ADA recommendation. HDL cholesterol of greater than 40mg/dl in men and 50mg/dl in women was seen in 274 (68.5%). LDLc of less than 100mg/dl was seen in 63.5% of the subjects. Finally, triglyceride level of < 150mg/dl (1.7mmol/l) was seen 76% of the study participants.

**Occurrence of the indices of microvascular complications (Table 2):** two hundred and seventy-seven (69.3%) had one or more microvascular complications of diabetic neuropathy (DN), diabetic nephropathy (D Nephropathy) and diabetic retinopathy (DR): DN 328 (82%), DR 184 (46%), D Nephropathy 176 (44%). The frequency of microvascular complications was higher in those with > 6 years DM duration.

**Table 2:** frequency of microvascular complications in participants

Variables	Total (%) N = 400	p- value	χ2	df
**Neuropathy**				
Present	338(82)	0.175	1.84	1
Absent	72(18)			
**RETINOPATHY**				
No apparent DR	217(54.25)	0.087	10.97	1
Diabetic Retinopathy	183(45.75)			
**NEPHROPATHY**				
Albumin in urine No -albuminuria	224(56)	0.938	3.07	1
Microalbuminuria	176(44)			

DR - diabetic retinopathy

### Occurrence of the indices of macrovascular complications (Table 3)

**Table 3:** distribution of macrovascular complications in participants

Variables	Total 400 (%)	p - value	df	χ2
**Peripheral arterial diseases intermittent claudication**				
Present	81(20.25)	0.104	1	2.64
Absent	319 (79.75)			
**ABI**				
Normal (>0.9)	230(57.5)	0.519	1	
Mild (0.7-0.9)	149(37.25)			
Moderate (0.4-0.69)	21(5.25)			
Severe (<0.4)	0(0)			
**Cerebrovascular diseases TIA**				
Present	5(1.25)	0.735	1	0.48
Absent	395(98.75)			
**Stroke**				
Present	16(4)	0.066	1	3.38
Absent	384(96)			
**ECG findings for IHD**				
Present	37(9.25)	0.753	1	0.10
Absent	363(90.75)			
**Central chest pain**				
Yes	4(1)	0.920	1	0.10
No	396(99)			

**ABI-** Ankle Brachial index**. IHD -** Ischaemic Heart Disease**, TIA -** Transient Ischaemic Attack**, PAD -** Peripherial ArteriaI Diseases

Of the 400 type 2 DM subjects studied, 196(49.0%) had one or more macrovascular complications.

i) For peripheral arterial disease (PAD), history of Intermittent claudication was obtained in 81(20.25%), abnormal ABI occurred among 170(42.5%) subjects: Mild (0.7-0.9)-149, Moderate (0.4-0.69) - 21, Severe (< 0.4) - 0.

ii) Cardiovascular disease - TIA in 5(1.25%) and Stroke 16(4%) in the study participants. Central chest pain was reported in 4(1.0%) of the study participants. Eletrocardiograghy (ECG) features of Ischaemic heart diseases was observed in 9.3% of the study subjects. The ECG features of IHD seen were mostly ST-segment depression of more than 1mm in more than one lead, and T-wave inversion. The most frequent ECG feature observed was LVH, which was observed in 88(22.0%) of the study subjects.

### Factors associated with diabetes complications (Table 4)

**Table 4:** factors associated with the presence of diabetes complications

Variable	Unadjusted ORs (95% CI)	P - value	Adjusted ORs (95% CI)	P - value
**Age, years**	1.05 (0.97 - 1.13)	0.250	1.18 (1.01 - 1.38)	0.038
**Sex**				
Male	2.29 (0.44 - 11.96)	0.325	5.39 (0.49 - 59.70)	0.170
**Smoking status**				
Present smoking	0.02 (0.00 - 0.28)	0.005	0.00 (0.00 - 0.07)	0.001
Past smoking	0.90 (0.10 - 7.87)	0.927	0.16 (0.01 - 2.84)	0.212
**Physical Activity**				
Physically active	0.68 (0.13 - 3.56)	0.649	0.51 (0.05 - 5.05)	0.565
**Diabetes Duration, years**	0.98 (0.87 - 1.11)	0.787	0.93 (0.80 - 1.08)	0.356
**Waist circumference, cm**	1.02 (0.95 - 1.10)	0.599	1.17 (1.00 - 1.36)	0.044
**Body Mass Index, Kg/m2**	0.96 (0.84 - 1.10)	0.611	0.77 (0.57 - 1.04)	0.91
**Fasting plasma glucose, mmol/L**	0.93 (0.79 - 1.10)	0.395	1.01 (0.77 - 1.33)	0.937
**Glycated Haemoglobin, %**	0.85 (0.63 - 1.14)	0.278	0.81 (0.51 - 1.27)	0.305

Three hundred and ninety-two (98%) has at least a complication of diabetes. Regression (univariate and multivariate) analyses were conducted to determine the factors assocaited with presence of diabetes complications. Advancing age aOR (1.18 [95%CI 1.01 - 1.38]) and waist circumference (aOR 1.17 [95% CI 1.00 - 1.36]) were the significant factors associated with diabetes complications after adjusting for sex, smoking status, physical activities and glycaemic status.

## Discussion

This is a descriptive cross-sectional study of diabetic complications in subjects with type 2 diabetes mellitus, aim to determine the pattern and frequency of Type 2 diabetic (T2DM) complications in an out- patient clinic. It also describes the distribution of indices of cardiometabolic risks, microvascular and macrovascular complications in T2DM subjects attending DM clinic in OAUTHC Ile- Ife, south West Nigeria. The study participants were in the age range 51- 70 years which is similar to a previous report [3,5]. Increasing age, increasing inactivity and longevity of diabetic patient due to improved care were attributed to be responsible for the increasing prevalence of Type 2 DM with age [1,3]. The history of cigarette smoking and alcohol consumption which are known risk factors for diabetes complications is consistent with the findings of previous studies [23,24]. There was overall high prevalence of overweight and obesity in the study participants as was noted previously in diabetics and non-diabetic Nigerians [25,26]. Most of the participants are traders and this is an occupation that tend to limit physical activity especially in females its resultant sedentary habits may contribute to the overweight and obesity. The prevalence of hypertension is high in the study participants; hypertension is a known risk factor for type 2 diabetes. The prevalence of hypertension in diabetes is variable worldwide and hypertension is an independent risks factor for microvascular and macrovascular disease. A multicenter study recently reported a prevalence of 60.9% [6]. The higher prevalence rate of 78% reported in our study compared with an earlier study in the same center may be due to the fact that this study shows the increasing prevalence with duration of both hypertension and diabetes with age [27]. Poor glycaemic control was seen in as many as 75.5% of the subjects. Probable reasons for poor control may include poor health seeking behavior, poverty, poor compliance and adherence with follow up visits and medications amongst others. Many people in our society also make use of alternative medicines like roots and herbs in treating their ailments, Factors identified for the poor glycaemic control in that study included poverty, illiteracy and poor compliance and adherence with medications [28]. This study showed a high occurrence of dyslipidaemia among study participants. This maybe attributable to more conservative use of lipid lowering therapy like statins noted (47.9% men and 43.8% women) in this study.

The prevalence of microvascular complications was high. Frequency of microvascular complication is higher in those with medium and long duration of diabetes than those with shorter duration of diabetes [29]. The observed prevalence of DM nephropathy of 44.0% this study is high and this might be due to poor glycaemic and blood pressure control in this study population. A study identified that diabetes subjects with diabetic nephropathy have a high-risk group for excess cardiovascular morbidity [30]. The prevalence of retinopathy of 46% seen in this study is higher compared to 15.1% reported by Erasmus *et al*. [31]. This might be due to increasing prevalence of DM, the duration of diabetes and poor glycaemic and blood pressure control observed in this present study. The frequency of diabetic neuropathy in the study participants was 82%, diabetic neuropathy is one of the frequent manifestations of diabetic microvascular complications. The reason could also be due to the poor glycaemic control seen these subjects, increase weight as many of our patients were overweight is also a known risk factor. This frequency of occurrence compares well with the rate of 60-100% estimated in developed countries [32]. The prevalence of macrovascular diseases was 49%. The risk factors for macrovascular complications in elevated total cholesterol and poor glycaemic control. The prevalence of IHD by ECG criteria in persons with type 2 diabetes in this study was 9.3%, this shows an increasing prevalence from previous report with suggestive ECG findings of IHD [33]. Also, the low prevalence (1%) of subjects who presented with typical angina pain may be due to autonomic neuropathy in the subjects. Frequency of occurrence of PAD is high, this might be due to higher prevalence of poor glycaemic control, blood pressure control and dyslipidaemia in this study. The UK prospective study that showed that hyperglycaemia, dyslipidaemia, smoking, and higher blood pressure were associated with the subsequent development of PAD [34]. In this study, advancing age and central obesity were the factors with significant association with complications of diabetes. This study is limited in that, radiological investigations, such as the computed tomography (CT) scan and Duplex ultrasound scan were not used in the evaluation for cerebrovascular and peripheral vascular diseases respectively. This could result in a relatively lower detection rates of these complications.

## Conclusion

The occurrence of both microvascular and macrovascular complications is high, coupled with high rates of obesity, hypertension, poor glycaemic control and elevated total cholesterol. Diabetic retinopathy and peripheral arterial disease are the commonest microvascular and macrovascular complications respectively.

### What is known about this topic

- The prevalence of diabetic complications is known globally.

### What this study adds

- The occurrence of these complications are still very high and sub optimal glycaemic control remains a challenge in an emerging economy;
- There is the need for healthcare workers to identify and intensify the treatment of these complications to reduce the morbidity and mortality of these patients.
